# Multiplex PCR Assay for Unequivocal Differentiation of Actinobacillus pleuropneumoniae Serovars 1 to 3, 5 to 8, 10, and 12

**DOI:** 10.1128/JCM.00685-14

**Published:** 2014-07

**Authors:** Janine T. Bossé, Yanwen Li, Øystein Angen, Lucy A. Weinert, Roy R. Chaudhuri, Matthew T. Holden, Susanna M. Williamson, Duncan J. Maskell, Alexander W. Tucker, Brendan W. Wren, Andrew N. Rycroft, Paul R. Langford

**Affiliations:** a Section of Paediatrics, Department of Medicine, Imperial College London, St. Mary's Campus, London, United Kingdom; b Norwegian Veterinary Institute, Oslo, Norway; c Department of Veterinary Medicine, University of Cambridge, Cambridge, United Kingdom; d The Wellcome Trust Sanger Institute, Wellcome Trust Genome Campus, Cambridge, United Kingdom; e Animal Health Veterinary Laboratories Agency (AHVLA), Rougham Hill, Suffolk, United Kingdom; f Department of Pathogen Molecular Biology, London School of Hygiene and Tropical Medicine, London, United Kingdom; g Department of Pathology and Pathogen Biology, The Royal Veterinary College, Hawkshead Campus, Hertfordshire, United Kingdom

## Abstract

An improved multiplex PCR, using redesigned primers targeting the serovar 3 capsule locus, which differentiates serovars 3, 6, and 8 Actinobacillus pleuropneumoniae isolates, is described. The new primers eliminate an aberrant serovar 3-indicative amplicon found in some serovar 6 clinical isolates. Furthermore, we have developed a new multiplex PCR for the detection of serovars 1 to 3, 5 to 8, 10, and 12 along with *apxIV*, thus extending the utility of this diagnostic PCR to cover a broader range of isolates.

## INTRODUCTION

Actinobacillus pleuropneumoniae is a major cause of morbidity and mortality due to respiratory disease in pigs and is responsible for substantial economic losses worldwide (1). There are 15 serovars of A. pleuropneumoniae (2), based on the presence of surface carbohydrates, principally capsules, with their prevalence varying between geographic regions. For example, serovars 1 and 5 are common in North and South America, and serovar 2 is common in Europe (reviewed in reference 3; see references therein), whereas these serovars are absent or rare in the United Kingdom (4). Differences in virulence and immunogenicity have been reported for different serovars (5), though within serovars, isolates tend to be clonal (6, 7). Accurate serotyping is essential for informed diagnosis, epidemiological investigation of outbreaks, and detection of the emergence of serovars previously not found, or rare, within a geographical region. The serovar of an isolate is classically determined using antibody-based tests (3). However, cross-reactivity is a major limitation and has been reported between serovars 1 and 9 (8), serovars 4 and 7 (9), and serovars 3, 6, and 8 (reviewed in reference 10). Thus, multiplex PCRs based on capsule loci have been developed, including those for serovars 2, 5, and 6 (11), 1, 2, and 8 (12), and 1, 7, and 12 (13). We have additionally devised a multiplex PCR that determines whether an isolate is serovar 3, 6, or 8 (10). The latter multiplex PCR amplifies a fragment of the A. pleuropneumoniae-specific *apxIV* gene and serovar 3-, 6-, and 8-specific sequences derived from the capsule loci. In recent years, we have observed in our collection (and others have reported [14]) a double- banding pattern in some isolates, so that it is not possible to distinguish between serovars 3 and 6. In this study, we (i) discerned the genetic reason for the aberrant serovar 3/6 double-banding pattern, (ii) developed a new serovar 3-6-8 PCR with modified serovar 3 primers that eliminate the aberrant pattern, and (iii) extended that PCR to include a further six serovars commonly found throughout the world. This includes the detection of serovar 10, for which a capsule locus-based PCR has not been described previously.

## MATERIALS AND METHODS

### Bacterial strains.

In this study, we used 15 reference A. pleuropneumoniae strains (see reference 10 for details), as well as a collection of 334 A. pleuropneumoniae clinical isolates from Denmark (*n* = 166), the United Kingdom (*n* = 154) (including 9 serovar 6 isolates that previously tested positive for serovars 3 and 6), Cyprus (*n* = 6), Switzerland (*n* = 6), and China (*n* = 2). All the isolates were previously typed serologically and/or by capsule-specific PCR. In addition, 31 strains of other porcine-associated bacterial species were tested as negative controls These included Actinobacillus suis (*n* = 5), Actinobacillus minor (*n* = 3), Actinobacillus porcitonsillarum (*n* = 3), Actinobacillus porcinus (*n* = 3), Actinobacillus indolicus (*n* = 3), Haemophilus parasuis (*n* = 5), Pasteurella multocida (*n* = 3), Bordetella bronchiseptica (*n* = 2), Mycoplasma hyopneumoniae (*n* = 3), and Streptococcus suis P1/7 (*n* = 1).

### Capsule regions of MIDG2472 and 405.

In this study, we have generated shotgun genome sequence data for the A. pleuropneumoniae serovar 6 United Kingdom clinical isolate MIDG2472, which shows the double-banding pattern using the 3-6-8 multiplex PCR previously described (10), and for the serovar 8 reference strain, 405. A shotgun genome sequence was generated from genomic DNA prepared using the Blood and Tissue DNeasy kit (Qiagen) per the manufacturer's instructions. Libraries were prepared from 5 μg genomic DNA by standard protocols (15, 16) and paired-end sequencing was performed at the Wellcome Trust Sanger Institute (Cambridge) on an Illumina HiSeq 2000 analyzer for 75 cycles. Draft genome sequences were assembled using VelvetOptimiser 2.2.0 (17) and capsule loci were identified using BLAST searches (18). The complete capsule loci for the other serovar strains (1, 2, 3, 5, 6, 10, and 12) were identified from the available whole-genome sequences (19–21).

### Multiplex PCRs.

**FIG 1:**
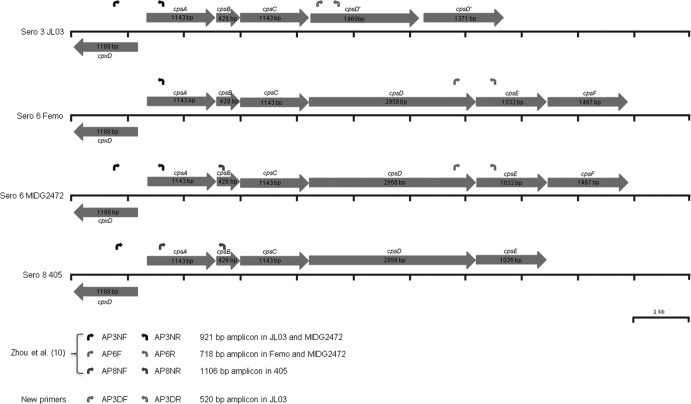
Location of primers within capsule loci of A. pleuropneumoniae serovar (Sero) 3, 6, and 8 strains. See Table 1 for oligonucleotide sequences of primers.

The primers used in this study are detailed in Table 1. The binding sites for the serovar 3, 6, and 8 primers in the respective capsule loci are shown in Fig. 1. Our original multiplex PCR for serovars 3, 6, and 8 was carried out as previously described (10). For the new multiplex PCR, the Qiagen Multiplex PCR Plus kit was used according to the manufacturer's instructions (Qiagen). Each reaction mixture contained 25 μl multiplex PCR master, 5 μl CoralLoad dye, 5 μl genomic DNA, 10 pairs of primers (each 10 pmol), and 13 μl water to a final volume of 50 μl. Genomic DNA extracted using the DNeasy blood and tissue kit (Qiagen) or present in boiled bacterial lysates prepared as described by Jessing et al. (11) was used as the DNA template for PCR amplification. Targeted gene amplification was initiated at 95°C for 5 min to activate the HotStar *Taq* plus DNA polymerase, followed by 25 cycles of 3-step cycling of denaturation at 95°C for 30 s, annealing at 61°C for 90 s, extension at 72°C for 2 min, and a final extension at 68°C for 15 min. PCR products were separated by 1.5% agarose gel electrophoresis, stained with ethidium bromide, and analyzed using a Gel Doc EI imager (Bio-Rad).

**TABLE 1:** Primers used in this study

Primer name	Sequence	Target gene^*a*^	Amplicon size (bp)	Reference or source
AP1F	CTGGAGTAATTACGGCGACTATTCC	*cps1B*	959	This study
AP1R	AGGAGAAGCTAGTAGTACTTGCATTTTC	*cps1B*		
AP2F	GAGTGTGATGATGATGCTCTGGTTC	*hyp*^*b*^	247	This study
AP2R	TACCAATAACTGTTGCAACTAACGC	*hyp*^*b*^		
AP3DF	CTATCATCTGTGCCAAGCTTACTACAC	*cps9D*′	520	This study
AP3DR	GGTTTAGAGGGGCAAATTGACTTG	*cps9D*′		
AP3NF	TTTGCGCTGTAGTGCTCCAAT	*cpxD*	921	10; not part of the 9-serovar PCR
AP3NR	AACAAATAAAGTTGCTCGAAAGTA	*cps2A*		
AP5F	AGCCACAAGACCCGAATGGTATAATG	*cps5B*	825	This study
AP5R	CCATCAAATGCAGCTTCAAGGAGC	*cps5B*		
AP6NF	AACCACTCACTTTCCACATTA	*cps6D*	718	10
AP6NR	AATCGGAAGGTTTTGGTCTCGTG	*cps6E*		
AP7F	TCTAGGTATTACTGGTGTTCCTGATG	*cps2D*	600	This study
AP7R	CGTCCAACACGAGCAACTACG	*cps2D*		
AP8NF	TTAGTTGCGCAAACGGCTTTTGAA	*cps8A*	1,106	10
AP8NR	GATTAAACTGGTCCGTCGAAATG	*cps8B*		
AP10F	GGTGGTGATGGAACAAGGTTATGG	*cps10A*	183	This study
AP10R	CTGTAATTGATGCGAAATAGTAGATTGGTGC	*cps10A*		
AP12F	TAAAGGTATTATAACGCCGGCTCT	*cps1A*	347	This study
AP12R	CTCCCATCTGTTGTCTAAGTAGTAG	*cps1A*		
apxIVA1	TTATCCGAACTTTGGTTTAGCC	*apxIV*	418	10
apxIVA3	CATATTTGATAAAACCATCCGTC	Intergenic^*c*^		
oAPXIVA-TSP1	CTTTCGCTCATGCGACTATG	*apxIV*	423	23; not part of the 9-serovar PCR
oAPXIVA-TSP2	TTTCCGTCCGGTTTATTCAG	*apxIV*		

a Capsule gene designations are those used by Xu et al. (20). There are some conserved gene names between different serovars with similar capsule types. However, primers were designed to bind strictly serovar-specific sequences within these genes.

b Serovar 2-specific open reading frame (ORF) immediately downstream of *cpsD* encoding a hypothetical protein.

c Intergenic region immediately downstream of *apxIV*.

### Nucleotide sequence accession numbers.

The DNA sequences of the capsule loci of strains MIDG2472 and 405 have been deposited in GenBank under accession numbers KJ685492 and KJ685493 , respectively.

## RESULTS AND DISCUSSION

**FIG 2:**
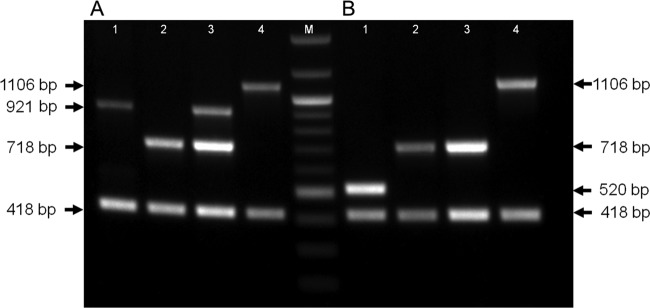
Comparison of original (10) and new (this study) multiplex PCRs for detection of A. pleuropneumoniae serovars 3, 6, and 8. (A) Original multiplex PCR for amplification of *apxIV* (418 bp), serotype 3 (921 bp), serotype 6 (718 bp), and serotype 8 (1,106 bp) amplicons. (B) New multiplex PCR for amplification of *apxIV* (418 bp), serotype 3 (520 bp), serotype 6 (718 bp), and serotype 8 (1,106 bp) amplicons. Lane M, 100-bp ladder; lanes 1, serovar 3, S1421; lanes 2, serovar 6, Femo; lanes 3, serovar 6, MIDG2472; lanes 4, serovar 8, 405.

**FIG 3:**
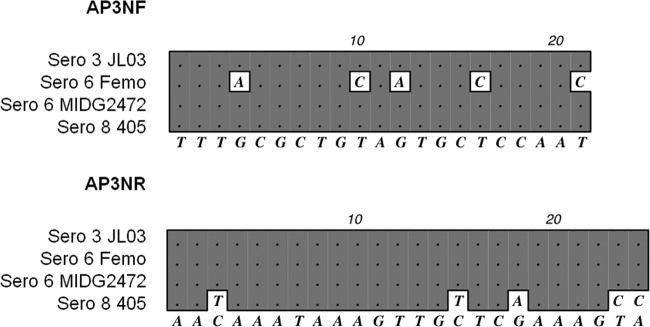
Alignment of the binding sites for primers AP3NF and AP3NR within the capsule loci of A. pleuropneumoniae serovar (Sero) 3, 6, and 8 strains. There are perfect binding sites for the AP3NF primer in the *cpxD* gene in strains JL03, MIDG2472, and 405. In strain Femo, the *cpxD* sequence differs slightly at the target site, such that the primer AP3NF does not bind. There are perfect binding sites for AP3NR in the *cpsA* gene in strains JL03, Femo, and MIDG2472. In strain 405, the *cpsA* sequence differs slightly at the target site, such that the primer AP3NR does not bind.

The first aim of this study was to determine the reason for the amplification of bands indicating serovars 3 and 6 in some serovar 6 clinical isolates. In our original multiplex PCR, the serovar 6-specific primers (AP6F and AP6R) were derived from a previously published PCR (11, 12). Their binding sites in the serovar 6 capsular locus are shown in Fig. 1. The serovar 3 primers (AP3NF and AP3NR) were designed to amplify a 921-bp fragment from *cps3A* (APJL_1614) to *cpxD* (APJL_1615), and the serovar 8 primers (AP8NF and AP8NR) were designed to amplify a 1,106-bp fragment from *cps8A* to *cps8B*, as indicated in Fig. 1 for the serovar 3 and serovar 8 capsule loci. Our original serovar 3 primers (AP3NF and AP3NR) were based on a DNA sequence that we obtained from a chromosome-walking strategy using serovar 3 reference strain S1421 prior to the availability of the serovar 3 JL03 whole-genome sequence (10, 22). AP3NF and AP3NR amplify a 921-bp PCR product in S1421 (serovar 3) but not the serovar 6 Femo reference strain (Fig. 2A, lanes 1 and 2), whereas MIDG2472 amplifies this 921-bp band as well as the serovar 6-indicative 718-bp product (Fig. 2A, lane 3). DNA sequence analysis of the capsule locus of MIDG2472 and Femo provides an explanation for the aberrant double-banding pattern. In MIDG2472, in addition to the serovar 6-specific region amplified by AP6F and AP6R, there are perfect binding sites for AP3NF and AP3NR (Fig. 1 and 3). In Femo, there is a binding site for AP3NR, but that for AP3NF is partially redundant (Fig. 3), such that, under the PCR conditions used, no amplification occurs. In strain 405 (serovar 8), the AP3NR binding sequence is partially redundant (Fig. 3), such that amplification with AP3NF does not occur. Thus, in Femo, only a 718-bp serovar 6-specific amplicon is present, while in MIDG2472 there is an additional 921-bp amplicon resulting from binding of the serovar 3 primer pair. In summary, the aberrant serovar 3/6 banding pattern results from the lack of specificity of the serovar 3 primers AP3NF and AP3NR in some serovar 6 isolates, such as MIDG2472. The two Japanese isolates with aberrant banding patterns were also found to have perfect priming sites for AP3NF and AP3NR (14). Thus, to eliminate the aberrant 3/6 banding pattern, we have reformulated our original 3-6-8 PCR by replacement of the serovar 3 primers. All other primers and PCR conditions were otherwise as described originally (10). The new serovar 3 PCR primers AP3DF and AP3DR are designed to amplify a 520-bp fragment of the serovar 3-specific APJL_1611 (*cpsD*) gene (Fig. 1). A comparison of old and new PCRs with the serovar 3, 6, and 8 reference strains and serovar 6 “aberrant” strain (MIDG2472) is shown in Fig. 2. The new PCR (Fig. 2B) eliminates the aberrant banding, there being amplicons at 520 bp, 718 bp, and 1,106 bp specific for serovar 3, 6, and 8 strains/isolates, respectively. For the unequivocal differentiation of serovar 3, 6, and 8 A. pleuropneumoniae isolates, we recommend that investigators use the modified PCR described in this study.

**FIG 4:**
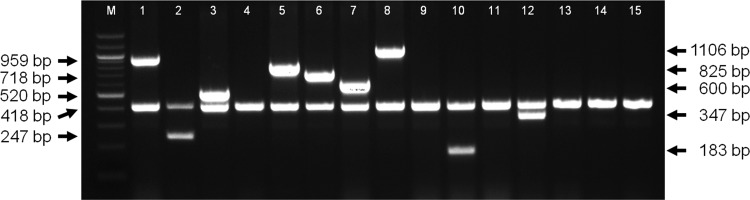
Multiplex PCR for detection of A. pleuropneumoniae serovars 1 to 3, 5 to 8, 10, and 12 and *apxIV*. An *apxIV* (418 bp) amplicon is detected in all 15 serovars, while serovar-specific amplicons are detected for serovar 1 (959 bp), serovar 2 (247 bp), serovar 3 (520 bp), serovar 5 (825 bp), serovar 6 (718 bp), serovar 7 (600 bp), serovar 8 (1,106 bp), serovar 10 (183 bp), and serovar 12 (347 bp). Lane M, 100-bp ladder; lane 1, 4074^T^; lane 2, S1536; lane 3, S1421; lane 4, M62; lane 5, L20; lane 6, Femo; lane 7, WF83; lane 8, 405; lane 9, CVJ13261; lane 10, D13039; lane 11, 56153; lane 12, 8329; lane 13, N-273; lane 14, 3906; lane 15, HS143.

Next, the utility of the new serovar 3-6-8 PCR was extended to include serovars 1, 2, 5, 7, 10, and 12, which are more prevalent in some countries, but which are either absent (as is the case for serovars 1 and 5) or are comparatively rare in the United Kingdom. Serovar-specific sequences were derived from whole-genome sequences: GenBank accession numbers ADOD00000000 (4074, serovar 1), ADOE00000000 (S1536, serovar 2), CP000687 (JL03, serovar 3), ADOF00000000 (M62 serovar 4), CP000569 (L20, serovar 5), ADOG00000000 (Femo, serovar 6), CP001091 (AP76, serovar 7), ADOI00000000 (CVJ13261, serovar 9), ADOJ00000000 (D13039, serovar 10), ADOK00000000 (56153, serovar 11), ADOL00000000 (1096, serovar 12), and ADOM00000000 (N273, serovar 13). The details of the primer pairs that were derived for use in the 9-serovar multiplex PCR are provided in Table 1. The 9-serovar multiplex PCR was initially tested against the A. pleuropneumoniae reference strains (Fig. 4). As expected, all of the reference strains had an *apxIV* amplicon of 418 bp, as reported previously (10), and appropriate serotype-specific amplicons of predicted sizes were detected for the reference strains of serovars 1 to 3, 5 to 8, 10, and 12. Subsequently, the PCR was evaluated using clinical A. pleuropneumoniae isolates, as well as other actinobacilli, other Pasteurellaceae, and other major pathogens of pigs. Additionally, virtual PCRs were carried out on available genomes of the species investigated, where available. A breakdown of the PCR results for clinical A. pleuropneumoniae isolates is shown in Table 2. Serovar designation of isolates had been carried out either by PCR, as described previously, or by antibody-based serotyping. All of the 334 clinical A. pleuropneumoniae strains tested amplified the predicted serovar amplicons. Five strains were positive for the serovar 1 amplicon but did not amplify the 418-bp *apxIV* band. These strains were subsequently tested for *apxIV* using the primers oAPXIVA-TSP1 and oAPXIVA-TSP2 (23), and each strain produced a 423-bp band indicating that *apxIV* is present (with no IS*Apl1* insertion). We recommend that where an isolate is strongly suspected to be A. pleuropneumoniae and there is a lack of either *apxIV* or a serovar-specific amplicon in our 9-serovar PCR, researchers retest the isolate with alternate A. pleuropneumoniae-specific primers such as those indicated above. If the isolate is confirmed as A. pleuropneumoniae, it is likely that its serovar is not covered by our multiplex PCR. While other multiplex PCRs have been reported for all but serovar 10, these have typically involved coverage of three serovars (see references 11–13). None have been described that include serovar 10 as described here. Serovar 10 isolates have been reported in Canada, Denmark, Germany, Hungary, Spain (3), and the United Kingdom (4), although not as one of the prevalent serovars.

**TABLE 2:** Number of serovar-specific and *apxIV* amplicons detected in 334 clinical isolates of A. pleuropneumoniae using the multiplex PCR

Serovar no.	No. of isolates	No. of specific amplicons for serovar no.:									No. of *apxIV* amplicons
		1	2	3	5	6	7	8	10	12	
1	21	21	0	0	0	0	0	0	0	0	16^*a*^
2	45	0	45	0	0	0	0	0	0	0	45
3	9	0	0	9	0	0	0	0	0	0	9
5	23	0	0	0	23	0	0	0	0	0	23
6	28	0	0	0	0	28	0	0	0	0	28
7	36	0	0	0	0	0	36	0	0	0	36
8	115	0	0	0	0	0	0	115	0	0	115
10	28	0	0	0	0	0	0	0	28	0	28
12	29	0	0	0	0	0	0	0	0	29	29

a See Results and Discussion in the text for discussion of the 5 isolates that were positive for the serovar 1 amplicon but did not amplify the 418-bp *apxIV* band.

The specificity of the multiplex PCR was further tested using 31 isolates representing porcine-associated bacterial species other than A. pleuropneumoniae, which were derived from our collection and have been assigned at the species level by conventional biochemical testing. None of these strains produced any amplicons with the new 9-serovar multiplex PCR.

In summary, we have developed a single-tube multiplex PCR that unequivocally differentiates A. pleuropneumoniae serovars 1 to 3, 5 to 8, 10, and 12. With the use of our well-characterized collection of A. pleuropneumoniae isolates and other porcine-associated bacterial species, the new PCR was 100% sensitive and specific for serotyping. In regard to the United Kingdom, the PCR covers all of the serovars (2, 3, 6, 7, 8, 10, and 12) that were previously reported (4), although a single serovar 9 isolate has subsequently be found (24).

The inclusion of *apxIV*-specific primers should allow for detection of A. pleuropneumoniae serovars not included in the multiplex PCR, though we have identified a small number (5/334) of strains that failed to produce the predicted 418-bp amplicon. In those strains, an *apxIV* band was obtained with alternate primers. Although we did not investigate further, the new multiplex PCR could be adapted to detect relevant serotypes found in different countries by excluding or including capsule-specific primers for other serovars as required.
